# Effects of receptor modification and temperature on dynamics of sensory complexes in *Escherichia coli *chemotaxis

**DOI:** 10.1186/1471-2180-11-222

**Published:** 2011-10-06

**Authors:** Sonja Schulmeister, Karin Grosse, Victor Sourjik

**Affiliations:** 1 Zentrum für Molekulare Biologie der Universität Heidelberg, DKFZ-ZMBH Alliance, Im Neuenheimer Feld 282, 69120 Heidelberg, Germany; 2 BioQuant, Im Neuenheimer Feld 267, 69120 Heidelberg, Germany

## Abstract

**Background:**

Extracellular stimuli in chemotaxis of *Escherichia coli *and other bacteria are processed by large clusters of sensory complexes. The stable core of these clusters is formed by transmembrane receptors, a kinase CheA, and an adaptor CheW, whereas adaptation enzymes CheR and CheB dynamically associate with the clusters via interactions with receptors and/or CheA. Several biochemical studies have indicated the dependence of the sensory complex stability on the adaptive modification state of receptors and/or on temperature, which may potentially allow environment-dependent tuning of its signalling properties. However, the extent of such regulation *in vivo *and its significance for chemotaxis remained unclear.

**Results:**

Here we used fluorescence recovery after photobleaching (FRAP) to confirm *in vivo *that the exchange of CheA and CheW shows a modest dependency on the level of receptor modification/activity. An even more dramatic effect was observed for the exchange kinetics of CheR and CheB, indicating that their association with clusters may depend on the ability to bind substrate sites on receptors and on the regulatory phosphorylation of CheB. In contrast, environmental temperature did not have a discernible effect on stability of the cluster core. Strain-specific loss of *E. coli *chemotaxis at high temperature could instead be explained by a heat-induced reduction in the chemotaxis protein levels. Nevertheless, high basal levels of chemotaxis and flagellar proteins in common wild type strains MG1655 and W3110 enabled these strains to maintain their chemotactic ability up to 42°C.

**Conclusions:**

Our results confirmed that clusters formed by less modified receptors are more dynamic, which can explain the previously observed adjustment of the chemotaxis response sensitivity according to the level of background stimulation. We further propose that the dependency of CheR exchange on the availability of unmethylated sites on receptors is important to improve the overall chemotaxis efficiency by suppressing molecular noise under conditions of high ligand concentrations. Moreover, the observed stability of the cluster core at high temperature is in line with the overall thermal robustness of the chemotaxis pathway and allows maintenance of chemotaxis up to 42°C in the common wild type strains of *E. coli*.

## Background

Chemotaxis enables motile bacterial cells to follow environmental chemical gradients, migrating towards higher concentrations of attractants while avoiding repellents. Despite some deviations in protein composition, all studied bacterial chemotaxis systems rely on a similar strategy of following chemical gradients, using the same conserved core of signaling proteins. The pathway in *Escherichia coli *is the best-studied model, see [1,2] for recent reviews. Sensing and processing of stimuli in bacterial chemotaxis is performed by complexes that consist of several attractant-specific chemoreceptors, a histidine kinase CheA, and an adaptor protein CheW. Attractant binding to the periplasmic part of a receptor rapidly inhibits CheA autophosphorylation, reducing phosphotransfer to the motor regulator CheY and thereby promoting smooth swimming. This initial rapid response is followed by slower adaptation, which is mediated by methylation of receptors on four specific glutamate residues by a methyltransferase CheR. The inverse reaction of receptor demethylation is mediated by the methylesterase CheB. Receptors are originally expressed in a half-modified state (QEQE), where glutamines (Q) mimic the effects of methylated glutamates and are deamidated by CheB. Higher modification of receptors increases activity of the associated CheA and lowers receptor sensitivity to attractants, thereby allowing cells to adapt to a persistent attractant stimulus [3-9]. The feedback from the sensory complex activity to the methylation system is believed to come primarily from the substrate specificity of adaptation enzymes, with CheR preferentially methylating inactive receptors and CheB preferentially demethylating active receptors [10-12]. An additional negative feedback is provided by the CheA-mediated phosphorylation of CheB, which increases CheB activity but is not essential for chemotaxis [13] and has little effect on the kinetics of adaptation to positive stimuli [10,14,15].

Although *in vitro *experiments suggest that the transmembrane signal transduction and kinase regulation in chemotaxis could be performed by small receptor-kinase complexes that consist of two to three receptor dimers, several CheW and one CheA molecules [11,16-18], in the cell the receptor-kinase sensory complexes are organized into macromolecular clusters that can contain thousands of receptors and associated chemotaxis proteins. Larger clusters typically localize at the cell poles, while several smaller clusters are found along the cell body [19-21]. In these clusters, receptors are arranged in roughly hexagonal arrays that are presumably formed by trimers of receptor homodimers [22-25], with different receptors able to form mixed trimers [26]. Clusters are further stabilized by the association of CheA and/or CheW [19,20,27-29]. Receptor clusters are important for signal processing in chemotaxis, whereby allosteric interactions between receptors within clusters allow amplification and integration of chemotactic signals [7,30-33]. All other chemotaxis proteins - CheR, CheB, CheY and CheZ - localize to receptor clusters in *E. coli *through association with either receptors (CheR) or CheA (CheZ and CheY) or both (CheB) [20,34-36]. Receptor clustering plays therefore an additional role by providing a scaffold for chemotaxis signalling [2]. The relatively stable signal-processing core of these clusters is composed of receptors, CheA, CheW and a phosphatase CheZ, along with the dynamically exchanging adaptation enzymes and CheY [37]. Adaptation enzymes are believed to primarily localize to the clusters via association with the C-terminal pentapeptide sequence of major receptors Tar and Tsr [35,36,38-40], but they also bind to their substrate sites - unmethylated glutamates for CheR and glutamines or methylated glutamates for CheB - on the receptors. Moreover, CheB also binds to the P2 domain of CheA, competing for the binding site with CheY [40,41].

The aim of this study was to investigate whether cluster stability *in vivo *is regulated by such physiologically relevant factors as adaptation to the chemotactic signals and by the environmental temperature. Several biochemical studies indicated that stability of sensory complexes might strongly increase with the level of receptor methylation [7,42]. However, a more recent study reported extreme ultrastability of the biochemically reconstituted sensory complexes with no discernible effect of receptor modification under the reference conditions [43], although complexes formed by the less modified receptors did show higher susceptibility to destabilizing agents. Surprisingly, this later study also reported a dramatic reduction of the complex stability at temperatures above 30°C. By performing an *in vivo *analysis of cluster stability using fluorescence recovery after photobleaching (FRAP), we were able to reconcile these apparently conflicting biochemical studies by showing that the exchange of CheA and CheW at receptor clusters is weakly dependent on the receptor modification. Our results also suggest a strong dependence of the exchange rates of adaptation enzymes on their ability to bind substrate sites on receptors, and on CheB phosphorylation. We propose that both effects play an important role in the overall strategy of bacterial chemotaxis. Moreover, in line with the recently described thermal robustness of the chemotaxis pathway [44] we observed that stability of the cluster signalling core is not affected by temperature and that the common wild type *E. coli *strains can perform chemotaxis up to 42°C.

## Results

### Receptor modification affects stability of the cluster core

To test effects of receptor modification on the exchange dynamics of CheW and CheA at receptor clusters, FRAP experiments were performed in an adaptation-deficient (Δ*cheRcheB*) strain and in the CheR^+ ^CheB^+ ^strain. In the former strain, receptors are present in their original half-modified (QEQE) state, which leads to a nearly maximal activation of the associated CheA *in vivo *[5,8,32]. In contrast, in the adapted CheR^+ ^CheB^+ ^strain the average level of receptor modification and activity are significantly lower [5,8,32,44] (see also additional file 1, Figure S1). To facilitate FRAP experiments, both strains carried an additional deletion of the negative regulator of late flagellar and chemotaxis gene expression, anti-sigma factor FlgM. This deletion leads to an approximately 6-fold overexpression of all chemotaxis genes and consequently to larger clusters, without any negative effects on chemotactic performance [37,45].

FRAP experiments were performed as previously described [37], whereby the fluorescence was bleached by two short laser pulses in the polar region of the cell, and subsequent recovery of relative fluorescence at the pole was followed over time (see *Methods *for details). As in this previous study, we used the C-terminal fusion of yellow fluorescent protein to CheW (CheW-YFP) and the N-terminal fusion to a truncated form of CheA that lacks first 258 amino acids (YFP-CheA^Δ258^). The latter fusion was chosen because it has a more clear localization pattern to receptor clusters than YFP fusion to the full-length CheA (CheA_L_) or the natively occurring short version of CheA (CheA_S_). Notably, all CheA fusions and both N- and C-terminal CheW fusions showed similar exchange kinetics in previous FRAP experiments, suggesting that the exchange kinetics at the cluster is unaffected by the YFP fusion [37]. Consistent with that, CheW-YFP fusion has been shown to form ultrastable ternary complexes *in vitro*, similar to those formed by the untagged CheW [43].

**Figure 1:**
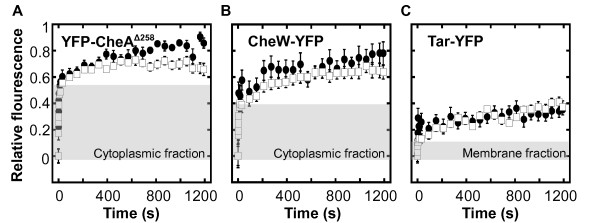
**Protein exchange at the cluster core**. **(a-b) **Recovery of YFP-CheA^Δ258 ^(a) and CheW-YFP (b) in strain LL4 (CheR^+ ^CheB^+^) where receptors are in the low modification state (filled circles) and in strain LL5 *(ΔcheR ΔcheB*) where receptors are in the intermediate modification state (white squares). **(c) **Recovery of unmodified Tar^EEEE^-YFP (filled circles) and fully modified Tar^QQQQ^-YFP (white squares) receptors in strain LL5. Curves represent means of 14 to 27 experiments, with error bars indicating standard errors. To reduce variability associated with the varying depth of bleaching, the value of the first post-bleach point was subtracted prior to normalization to the relative intensity before photobleaching (see *Methods*). Grey shading indicates the initial rapid recovery of the fusion protein that is not incorporated into the cluster and freely diffuses in the cytoplasm or in the plasma membrane (see text).

Thus obtained recovery kinetics was clearly biphasic for all fusions (Figure 1). Our previous detailed analysis of FRAP data demonstrated that the initial phase of fast recovery corresponds to the exchange of the freely diffusing fusion protein in the region of interest, whereas the second phase specifically reflects protein exchange at the cluster [37]. In this study, we therefore were only interested in this slow phase of recovery, which reflects the concentration-independent rate constant of protein dissociation from the cluster (*k_off_*) [37]. Analysis of obtained recovery kinetics showed that the exchange of CheA, and to a lesser extent of CheW, was slower in Δ*cheRcheB *strain than in the CheR^+ ^CheB^+ ^strain (Figure 1a, b). Whereas in the CheR^+ ^CheB^+ ^strain the characteristic turnover time (*k_off_*^-1^) of CheA at the cluster was ~15 min, as observed before [37], little recovery was observed in the Δ*cheRcheB *strain even after 20 min. This strongly suggests that receptors with higher levels of modification (and therefore higher activity) form signalling complexes that are more stable.

To further test whether the level of modification directly affects the exchange of receptors at the cluster, we performed FRAP experiments on YFP fusions with two extreme modification states of an aspartate receptor Tar - fully unmodified Tar^EEEE ^and fully modified Tar^QQQQ^. These fusions were tested in Δ*cheRcheB *background, which also expresses the original untagged receptors in the half-modified state. This was necessary because YFP-tagged receptors do not form clusters very efficiently when expressed alone, presumably due to perturbing effects of multiple fluorescent proteins on the cluster structure. Little exchange was observed in this experiment even for the fully unmodified receptors (Figure 1c), suggesting that even inactive receptors are stably incorporated into the receptor clusters. The faster exchange of CheA at the clusters of less modified receptors is therefore likely to reflect the dynamics of kinase association with receptors rather than the exchange of receptors themselves.

### Receptor modification and pathway activity affect exchange of adaptation enzymes

**Figure 2:**
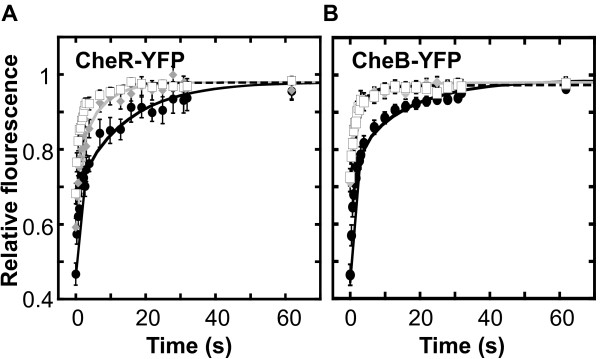
**Exchange kinetics of adaptation enzymes**. **(a) **Recovery kinetics of CheR-YFP in strain VS102 (CheR^+ ^CheB^+^) with receptors in low methylated state (filled circles, solid black line; data taken from [37]) and in strain LL5 that lacks chromosomal CheR and CheB (white squares, dashed black line), and recovery kinetics of YFP-CheR^D154A ^(gray diamonds, gray line) in strain LL5. **(b) **Recovery kinetics of CheB-YFP in strain VS102 (filled circles, solid black line, data taken from [37]), and of CheB^S164C^-YFP (gray diamonds, gray line) and CheB^D56E^-YFP (white squares, dashed black line) in LL5. Curves represent means of 13 to 30 experiments, with error bars indicating standard errors.

We next investigated whether the dynamics of the adaptation enzymes at the cluster might be regulated at the level of the receptor modification and/or the pathway activity. As mentioned above, CheR and CheB bind not only to the C-terminal pentapeptide sequence of receptors but also to their substrate sites on receptors - unmethylated glutamates and glutamines or methylated glutamates, respectively. Consistent with the significant contribution of the binding of CheR and CheB to their substrate sites to the overall exchange dynamics, we observed a clear increase in the exchange rates of CheR (Figure 2a) and CheB (Figure 2b) in strains where this binding was compromised. Whereas the characteristic exchange time of CheR in CheR^+ ^CheB^+ ^cells was ~15 sec, this time was reduced to ~6 sec in the strain that lacks *cheB*, thus having all receptors in a fully modified state (i.e., QEmQEm, where Em is the methylated glutamate), with no substrate sites available for methylation (Figure 2a and Figure S1a). A very similar reduction has been observed for the catalytic mutant of CheR (CheR^D154A^, [36]) in Δ*cheRcheB *cells (Figure 2a). Although in these cells receptors are in the half-modified (QEQE; Figure S1a) state and thus have available substrate sites, the catalytic mutant of CheR apparently fails to bind to these sites efficiently. The dependence of CheR exchange on the level of receptor modification is thus likely to be a direct consequence of its binding to the substrate sites, although it is still possible that receptor modification has an indirect, allosteric effect on the affinity of CheR binding.

Similarly, the characteristic exchange time for CheB was reduced from ~16 sec to ~4 sec upon mutation of the catalytic site (CheB^S164C^, [46]; Figure 2b), suggesting that the binding to the substrate sites is similarly important for the overall stability of CheB association with the cluster. A similar reduction in the exchange time, to ~2.5 sec, was observed upon mutating the phosphorylation site of CheB (CheB^D56E^; Figure 2b), consistent with a previous observation that unphosphorylated CheB shows weaker binding to receptor clusters [40]. Surprisingly, the exchange rate of the wild type CheB in the *cheR *background was similar to that in the CheR^+ ^CheB^+ ^strain (data not shown). We observed, however, that receptors were not fully deamidated in this strain (Figure S1b), likely providing sufficient number of substrate binding sites (Qs) for CheB molecules.

### In vivo *stability of the cluster core is not affected by temperature*

**Figure 3:**
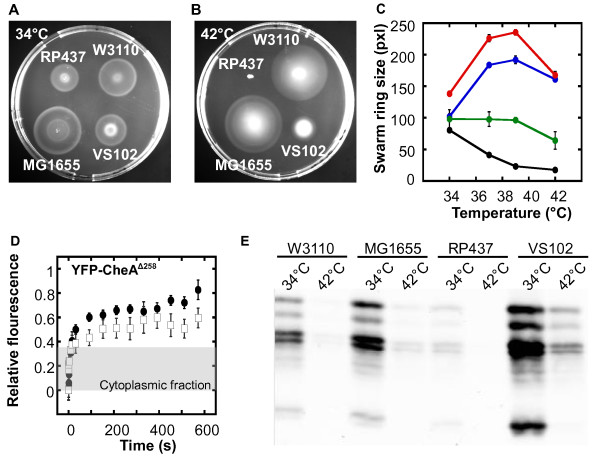
**Effects of temperature on chemotaxis and cluster stability**. **(a-b) **Effects of incubation temperature on swarming ability of *E. coli *strains. Representative swarm plates show swarm rings formed by indicated strains at 34°C (a) and 42°C (b) after 5 hours. **(c) **Corresponding swarming efficiency at a function of temperature for strains RP437 (filled circles), W3110 (white squares) and MG1655 (white circles). Standard errors are indicated. **(d) **Exchange of YFP-CheA^Δ258 ^at receptor clusters in strain VS102 at 20°C (filled circles, data from [37]) and at 39°C (white squares). Means of 10 to 20 experiments are shown. Error bars represent standard errors. Grey shading is as in Figure 1. **(e) **Temperature effects of expression levels of chemotaxis proteins, represented here by chemoreceptors. Expression was detected by immunoblotting as described in *Methods *using αTar antibody that also recognizes well other chemoreceptors. In CheR^+ ^CheB^+ ^strains used here, each receptor runs as several bands corresponding to different states of modification. See Figure S1 for assignment of individual bands.

Finally, we have analyzed effects of temperature on stability of the cluster core. *E. coli *K-12 strain RP437, used as a wild type in most studies of chemotaxis, as well as its parent strain B275, are known to lose their motility and chemotaxis above 37°C (Figure 3a-c) [47]. It was recently proposed that temperature sensitivity of chemotaxis may be related to the observed low stability of biochemically reconstituted chemosensory complexes at high temperature [43]. However, we observed that common wild type *E. coli *K-12 strains MG1655 and W3110 remain chemotactic up to 42°C (Figure 3a-c), despite having the same chemotaxis machinery as RP437. Consistent with that, the intracellular stability of receptor clusters, accessed by the dynamics of CheA exchange, showed no apparent decrease in stability at high temperature (Figure 3d).

These results suggest the downregulation of the chemotaxis gene expression as the most likely cause of the chemotaxis loss in RP437 at high temperature, consistent with the originally favoured explanation [47]. Indeed, under our growth conditions the expression of both major chemoreceptors, Tar and Tsr, was at least 10 times lower at 42°C than at 34°C (Figure 3e), which is likely to reflect a general temperature effect on expression of all chemotaxis and flagellar genes in *E. coli*. Notably, a similar reduction in the receptor levels was observed in all strains, demonstrating that the effect is not specific to the RP437-related strains. However, since the levels of chemotaxis proteins are generally much higher in MG1655 and W3110, these strains can apparently maintain sufficient expression even at 42°C, whereas protein levels in RP437 readily drop below the level that is necessary for chemotaxis [37,45]. This explanation is further supported by the observation that a substantial degree of chemotaxis was retained at 42°C in the RP437-derived *ΔflgM *strain VS102, which has elevated levels of all chemotaxis proteins (Figure 3e). Nevertheless, the improvement of chemotaxis at high temperatures was less pronounced in VS102 than in MG1655 and W3110, despite comparatively higher chemotaxis protein levels in the former strain. This suggests that the effect of high temperature cannot be solely compensated by the overexpression of chemotaxis proteins, probably because the low expression of early flagellar proteins, which are not upregulated in VS102, becomes limiting in this case.

## Discussion

Stimulation-dependent regulation of assembly and stability of sensory complexes can be important in signalling, and many signal transduction pathways in eukaryotes are regulated on this level [48]. Here we show that protein exchange at the sensory complexes in *E. coli *chemotaxis is affected by the signalling state of the pathway on many levels. First, stability of the sensory receptor-kinase core is higher for complexes formed by receptors that are in a higher modification state and consequently are more active. Such dependence is generally consistent with previous biochemical experiments [7,42], with lower structural stability of less modified receptors [49], and also with higher sensitivity of sensory complexes that are formed *in vitro *by the less modified receptors to destabilizing factors such as high pH or low ionic strength [43]. Our data also agree with *in vivo *studies that reported an increase in protein localization to the chemoreceptor clusters [50,51] at higher levels of receptor modification or activity. However, the effect *in vivo *is rather modest, and the observed regulation of complex stability dependent on receptor modification is unlikely to be directly involved in signal transduction. Rather, it may play a role in the adjustment of the signalling properties of receptor clusters, and can indeed explain the previously observed increase in the strength of cooperative receptor interactions within clusters upon increase in receptor modification [5]. Since increased methylation results from adaptation to increasing concentration of ambient attractant, higher stability and cooperativity within clusters can enhance the gain of the chemotaxis system at higher levels of ambient ligands, to closely follow physical limits of sensitivity posed by the noise in ligand binding [5].

The regulation of exchange at the cluster that was observed for the adaptation enzymes may be of even greater physiological significance. When CheR is unable to bind its substrate sites on the receptor, whether due to the mutation in the catalytic site of CheR or lack of unmethylated glutamates, the turnover was greatly accelerated. This suggests that the overall rate of CheR dissociation from receptors (*k_off_*) largely depends on its binding to the substrate sites, although such dependence remains to be confirmed by direct biochemical measurements. In principle, the level of receptor modification might also affect this turnover indirectly, through an allosteric regulation of CheR association with the C-terminal pentapeptide sequence of receptors. Regardless of the detailed molecular mechanism of such methylation-dependent acceleration of CheR exchange, we propose that faster turnover can increase the efficiency of adaptation by limiting the amount of time CheR spends in an unproductive association with a receptor molecule that cannot be further modified. This is particularly important for adaptation to high levels of ambient stimulus, when the kinetics and precision of adaptation become severely limited by the shortage of the free methylation sites [15,52].

Another important effect of the faster turnover of CheR at the cluster may be to specifically reduce the noise in the signalling output at increased levels of receptor methylation. Previous studies suggested that the level of phosphorylated CheY in adapted *E. coli *cells can vary substantially on the time scale of tens of seconds [53]. This can be explained by stochastic fluctuations in the number of cluster-associated CheR molecules [53-55] that would translate into the variable level of receptor methylation and ultimately into fluctuations of the activity of the pathway. Such fluctuations are expected to result in *E. coli *cells occasionally undertaking very long runs, enhancing the overall efficiency of the population spread through the environment in the search of chemoattractant gradients [54,55]. However, fluctuating levels of CheY-P are also predicted to severely impair the ability of bacteria to precisely accumulate at the source of the chemoattractant gradient, posing a trade-off dilemma for the chemotaxis strategy [55]. We propose that the observed increase in the turnover of CheR at the highly methylated receptors will specifically decrease noise in the pathway output for cells that have already reached high attractant concentration along the gradient, enabling them to efficiently accumulate at the source of attractant. The observed regulation of CheR exchange may therefore be an evolutionary selected trait that increases overall chemotaxis efficiency.

An acceleration of exchange was also observed for the catalytic mutant of CheB. This indicates that the CheB exchange is dependent on its binding to substrate sites, similar to CheR, though the molecular details of this effect remain to be clarified. Moreover, CheB exchange was strongly stimulated by mutating the phosphorylation site in the regulatory domain, which prevents CheB activation by phosphorylation. This latter effect confirms that the binding of CheB to receptor clusters is strengthened by phosphorylation, which may provide an additional regulatory feedback to the chemotaxis system ([40]; Markus Kollmann, personal communication).

Finally, we analyzed here the effects of temperature and showed that the thermal stability of the cluster core in the cell, determined by the exchange of CheA, is much higher than that of the biochemically reconstituted complexes [43]. While the factors responsible for the observed differences between stability of the sensory complexes *in vivo *and *in vitro *remain to be identified, such differences may be due to the active process of assembly and/or disassembly of the sensory complexes by specialized cellular chaperones as previously proposed [43]. In any case, thermal stability of the cluster core may be an important component of the overall thermal robustness of the chemotaxis pathway [44]. Consistent with that, the deterioration of chemotaxis in some *E. coli *strains above 37°C is apparently caused by the reduced expression of chemotaxis and flagellar genes rather than by the malfunction of the pathway. Moreover, although the observed effect of temperature on gene expression was not strain-specific, chemotaxis of the wild type strains MG1655 and W3110 was significantly less affected than chemotaxis of RP437. This difference was apparently due to the generally higher expression of chemotaxis proteins in MG1655 or W3110, which enables these strains to maintain expression that is sufficient for chemotaxis up to 42°C. Thus, the ability to maintain chemotaxis at high temperature is likely to be accomplished by a combination of the thermally robust pathway design [44] with the high thermal stability of chemosensory complexes and high basal expression levels of chemotaxis and flagellar proteins.

## Conclusions

**Figure 4:**
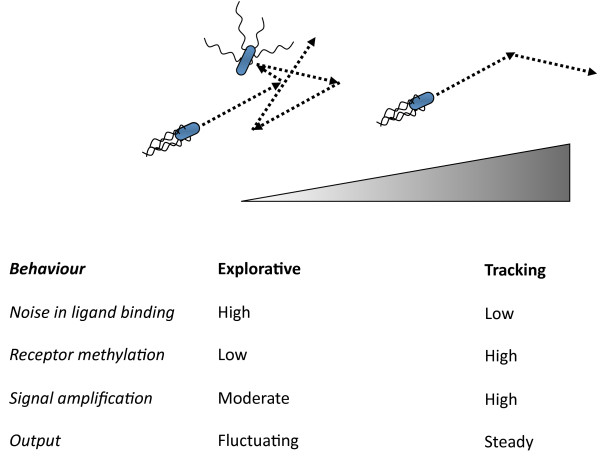
**Two regimes of bacterial chemotaxis behaviour and their characteristic features**. Attractant gradient is indicated. At low concentrations or absence of attractant the behaviour is explorative, while at high concentrations of attractant the behaviour is tracking. See text for details.

In summary, we observed that the rate of protein exchange at the chemosensory clusters in *E. coli *depends on the level of adaptive receptor modification. We believe that this dependency may reflect a specific regulatory mechanism to adjust the signalling properties of the chemotaxis system according to varying levels of ambient attractant stimulation, corresponding to two distinct regimes of bacterial chemotaxis that can be described as "searching" and "tracking" behaviour (Figure 4). Searching behaviour is exhibited by chemotactic bacteria when they explore the environment in the search of attractant gradients in the absence (or at low levels) of ambient ligand. In this regime the level of receptor modification is low, which would result in higher dynamics of the cluster core and slow exchange of CheR at the receptor clusters. The former apparently limits the cooperative interactions between receptors and consequently signal amplification by the clusters. This is physiologically meaningful because sensitivity towards small changes in attractant concentration under these conditions is physically limited by the stochastic noise in ligand binding. The long dwell time of CheR at receptors is also favourable for the explorative behaviour in this regime, because it produces large stochastic fluctuations in the pathway activity over time, thereby promoting faster spread through the environment. The second regime, tracking behaviour, is expected to occur when the cells are moving along the gradient and are already adapted to high ambient concentration of attractant. In that case, the levels of receptor methylation are high, which would increase the dynamics of CheR at the clusters, thereby effectively suppressing the stochasticity of the pathway output and allowing cells to follow the gradient more precisely. High receptor methylation would also enhance cluster stability, leading to stronger amplification of signals under conditions of the reduced ligand binding noise at high concentrations of ambient attractant.

Finally, we demonstrated the thermal stability of the chemosensory complexes *in vivo*, which may be an important component of the overall thermal robustness of the chemotaxis pathway [44]. This is consistent with the ability of the common wild type *E. coli *strains to chemotax efficiently up to 42°C. In these strains, the reduction of the chemotaxis and flagellar gene expression at high temperature is further balanced by high basal levels of the respective proteins, thus ensuring that chemotaxis is supported throughout the entire physiological range of temperatures.

## Methods

### Bacterial strains and plasmids

All strains used for FRAP measurements are derivatives of the *E. coli *K-12 strain RP437 that is conventionally used as the wild type for chemotaxis studies [56]: VS102 carries a deletion of the anti-sigma-factor *flgM*, resulting in an approximately 6-fold over-expression of all chemotaxis proteins [45], which has been previously shown to facilitate FRAP measurements of chemotaxis clusters in *E. coli *[37]. LL4 (*ΔflgM ΔcheY-cheZ*) and LL5 (*ΔflgM ΔcheR-cheZ) *served as backgrounds corresponding to receptors in low- and intermediate modification state, respectively. In addition, common *E. coli *K-12 wild type strains MG1655 and W3110 were used as controls for studies of temperature effects on chemotaxis. YFP tagged chemotaxis proteins were expressed from the vector plasmid pTrc99a (Amp^r^) under control of an isopropyl-β-D-thiogalactopyranoside (IPTG) inducible pTrc promoter [57]. Site-specific mutagenesis was used to introduce mutations into catalytic sites of CheR and CheB [36,40,46]. As reported previously, YFP fusions to CheR and CheB are fully functional, whereas fusions to CheA and CheW do not efficiently support chemotaxis but show proper localization to receptor clusters and interactions with their respective binding partners [36,40,46]. All expression constructs for the YFP fusions and respective induction levels employed for FRAP are presented in Table 1.

**Table 1:** Plasmids used in this study

Plasmid	Relevant genotype	Induction level (μM IPTG)	Reference
pDK54	*cheW-eyfp*	50	[37]
pSS8	*eyfp-cheA^Δ258^*	50	[37]
pDK137	*tar^EEEE^-eyfp*	20	*gift of David Kentner*
pDK138	*tar^QQQQ^-eyfp*	20	*gift of David Kentner*
pVS138	*cheB-eyfp*	100	[58]
pDK19	*cheR-eyfp*	100	[37]
pDK159	*cheB^S164C^-eyfp*	50	[40]
pDK183	*cheB^D56E^-eyfp*	50	*gift of David Kentner*
pDK116	*eyfp-cheR^D154A^*	50	[40]

### Cell growth and preparation

For FRAP measurements cells were grown as described elsewhere [37]. In brief, overnight cultures were diluted 1:100 in 10 ml TB (10 g/l tryptone, 5 g/l NaCl, pH 7.0) containing appropriate antibiotics and inducers (Table 1). After growing at 34°C with 275 rpm to OD_600_≈0.45-0.5 cells were two times washed in tethering buffer (10 mM KH_2_PO_4_/K_2_HPO_4_, 0.1 mM EDTA, 10 mM sodium lactate, 67 mM NaCl, 1 μM methionine, pH 7.0). To minimize growth and protein production, cells were subsequently incubated for at least 1 h at 4°C.

### FRAP Analyses and data processing

For FRAP experiments cells were immobilized on (poly)L-lysine-coated coverslips for 5 min. Measurements were usually performed at 20°C (RT) or when indicated at 39°C. For that, slides were placed in a metal chamber connected to a water bath. Cells were visualized with the 63× oil objective of a laser-scanning confocal microscope (Leica TCS SP2). Fluorescent cells were scanned by the 514 nm laser line of a 20 mW argon laser with 1-5% intensity and detected within 525-650 nm at 32-fold magnification. Regions of interest (ROIs) were bleached with two 0.336 s laser scans at 50% laser intensity using the same laser line. The following image series were recorded (Leica Confocal software, Version 2.61) by bidirectional scanning: one prebleach- and 10 postbleach images every 0.336 s, 10 postbleach images every 3 s and depending on protein 10-40 postbleach images every 30 s.

Images were analyzed by using a custom-written plug-in [37] for ImageJ software, Version 1.34l (W. Rasband, National Institutes of Health, Bethesda, MD; http://rsb.info.nih.gov/ij). For FRAP evaluation, the polar region was defined as 52 pixles, which is approximately 20% of the average cell length. Fluorescence of the ROI was normalized two times: first to the fluorescence of the entire cell in the same image to compensate for gradual bleaching during scanning, second to the prebleach value of the ROI, to make different experiments comparable. To reduce variability that arises due to varying depth of bleaching, for experiments shown in Figure 1 and 3d the value of the first post-bleach point was additionally subtracted and the curves were renormalized. Data were processed using KalaidaGraph software, Version 3.6 (Synergy Software).

For data fitting in Figure 2, protein exchange at chemotaxis clusters can be treated as a combination of anomalous diffusion and an exponential decay with the characteristic exchange time *τ_obs _*and fit with the following equation:

$$I\left(t\right)=\frac{F_{0}+F_{\infty }{\left(\frac{t}{t_{1∕2}}\right)}^{\alpha }}{1+{\left(\frac{t}{t_{1∕2}}\right)}^{\alpha }}+C\left(1-e^{-\left[\frac{t}{\tau_{obs}}\right]}\right),$$

where *F*_0 _accounts for the relative fluorescence intensity of free fluorescent protein after bleaching, *F*_∞ _is the corresponding intensity after recovery, *t_1/2 _*is half-time of recovery, α is the factor accounting for anomalous diffusion and *C *is the relative steady-state concentration of cluster-bound fluorescent protein [37]. Values for *t_1/2 _*and α has been taken from a previous study, where diffusion of the same proteins were measured in a strain without chemotaxis clusters [37].

### Soft agar chemotaxis assays

To test chemotaxis-driven spreading of MG1655, W3110 and RP437 on soft agar plates, 3 μl of an overnight culture grown in TB were dropped on soft agar plates (TB, 0.3% agar) and incubated for 5 hours at either 34°C, 37°C, 39°C or 42°C. Pictures were taken, swarm ring diameters were analyzed by ImageJ software and plotted using KalaidaGraph software.

### Immunoblotting

Immunoblotting was performed as previously described [44]. Cells were grown as described above to give the same OD_600 _for all strains, washed and collected by centrifugation, resuspedend in Laemmli buffer and lysed for 10 min at 95°C. Samples were separated on the 8% SDS-polyacrylamide gel and analyzed using primary polyclonal αTar antibody at 1:5,000 dilution and IRDye 800 conjugated secondary antibody (Rockland) at 1:10,000 dilution. Note that αTar antibody, which was raised against conserved signaling domain of receptor, recognizes other chemoreceptors with similar specificity. Membranes were scanned with an Odyssey Imager (LI-COR).

## Authors' contributions

SS and KG performed all experiments and participated in the design of the study and in writing the manuscript. VS conceived the study, participated in its design and wrote the manuscript. All authors read and approved the final manuscript.

## Supplementary Material

Additional file 1 **Figure S1. Modification levels of chemoreceptors in strains used for FRAP**. The figure shows levels of chemoreceptor modification in strains expressing CheR and CheB fusions, determined by immunoblotting with receptor-specific antibodies. Click here for file
