# Supplementary material for: Effects of receptor modification and temperature on dynamics of sensory complexes in Escherichia coli chemotaxis
Source: BMC Microbiol. 2011 Oct 6;11:222. doi: 10.1186/1471-2180-11-222 (PMC3203854; doi:10.1186/1471-2180-11-222)
Supplement: Additional file 1 — Figure S1. Modification levels of chemoreceptors in strains used for FRAP. The figure shows levels of chemoreceptor modification in strains expressing CheR and CheB fusions, determined by immunoblotting with receptor-specific antibodies. [file 1471-2180-11-222-S1.PDF]

Supplementary Figure S1

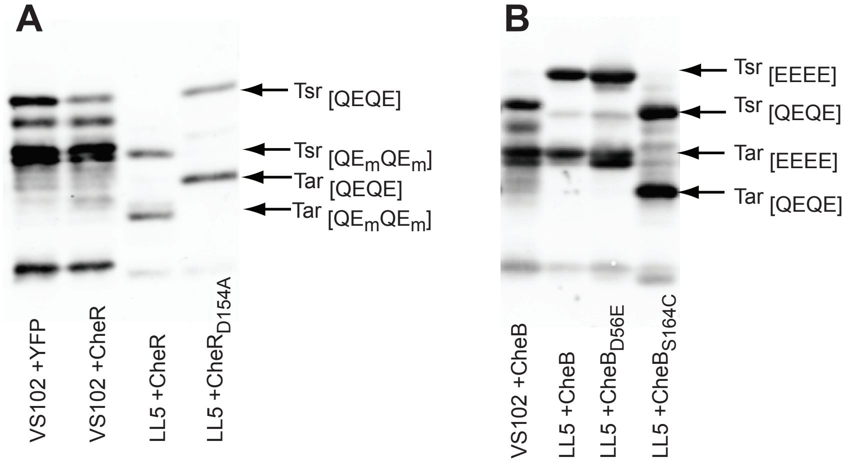

Modification levels of chemoreceptors in strains used for FRAP (Fig. 1 and Fig. 2). Receptors were separated according to their levels of modification on the SDS-polyacrylamide gel as described in Methods and detected using immunoblotting with anti-Tar antibody that also recognizes well other chemoreceptors. Arrows indicate major receptors Tar and Tsr and their modification states, whereby glutamine (Q) has similar effect on the function and mobility of receptors as methylated glutamate (Em). The band below Tar is likely to correspond to the minor receptor Aer.
